# Usage Data and Scientific Impact of the Prospectively Established Fluid Bioresources at the Hospital-Based MedUni Wien Biobank

**DOI:** 10.1089/bio.2018.0032

**Published:** 2018-12-17

**Authors:** Helmuth Haslacher, Marlene Gerner, Philipp Hofer, Andreas Jurkowitsch, Johannes Hainfellner, Renate Kain, Oswald F. Wagner, Thomas Perkmann

**Affiliations:** ^1^ Department of Laboratory Medicine and Medical University of Vienna, Vienna, Austria.; ^2^ Department of Pathology, Medical University of Vienna, Vienna, Austria.; ^3^ Institute of Neurology, Medical University of Vienna, Vienna, Austria.

**Keywords:** biobank, scientific output, access rate, biobank output, traceability

## Abstract

***Background and Aim:*** It is increasingly recognized that biomedical research has serious reproducibility issues, which could be overcome at least in part by standardized processing of biomaterials. Therefore, professional biobanks have emerged, positively influencing sample and data quality. However, quantitative data about a biobank's contribution to published results are still hard to find, although they could serve as valuable benchmark figures for the community. We therefore aimed to report usage data from the MedUni Wien Biobank facility regarding its prospective fluid cohorts.

***Methods:*** Input and access statistics and publication output were reported for the years 2010–2017. Performance dynamics were tested by correlation analyses according to Spearman. Additionally, virtual costs per sample were calculated.

***Results:*** The amount of annually collected aliquots rose significantly from 68,500 in 2010 to 151,966 in 2017 (*p* = 0.015), although no further increase was recorded after 2012 (*p* = 0.266). In the same period, the quotient of requested to stored aliquots increased from 3.5% to 6.1% (*p* = 0.001), as the yearly number of requested aliquots nearly quadrupled from 2401 to 9342. Likewise, the number of published research articles per year to which the MedUni Wien Biobank contributed increased from 2 (total impact factor: 8.6) in 2010 to 16 (total impact factor: 69.0) in 2017, resulting in a total of 69 identified publications. Currently, the biobank operates at 15- to 20-fold overproduction, leading to virtual costs per accessed sample of ∼€20.

***Conclusion:*** The reported usage data might serve as a benchmark for other hospital-integrated biobanks, and implies that academic biobanks are able to produce considerable scientific impact at comparable moderate costs.

## Introduction

It is increasingly recognized that poor reproducibility of results in biomedical research is to a relevant degree caused by low quality materials, leading to an annual loss of ∼$28 billion in the United States alone.^1^ Consequently, numerous biobanks have emerged to address the need for biomaterial and data produced under standardized conditions, most of them under public or academic control.^2^ However, despite their undisputed contribution to research, the actual degree to which biobanks facilitate research output is still unclear. Hence, comparative data from already established biobanks are highly warranted.

In 2006, the Medical University of Vienna embarked on a centralized biobanking strategy when the former Department of Medical and Chemical Laboratory Diagnostics (now: Department of Laboratory Medicine) began to assist clinical researchers in preanalytical sample handling and storage. To this end, an internal sample shipment and receiving process was designed in such a way that it could be easily integrated into routine preanalytical procedures. Following the example of disease-specific research biobanks,^2^ clinicians were invited to send biomaterial to this centralized facility. Using this approach, the biobank has established >70 different collections including ∼2 million fluid sample aliquots. However, the actual contribution of the MedUni Wien Biobank to biomedical research has yet to be evaluated, although the emerging data could serve as good comparative figures for comparable hospital-based biobanks.

We thus aimed to report its usage data and to draw implications for academic, hospital-based biobanking. Moreover, better traceability of resources in a published article is of great interest for the biobank, compilation of performance indicators,^3^ and the researcher, since the disclosure of resources used enhances data reliability.^4^ Hence, the present article aims moreover to expound the current protocols for liquid sample processing, storage, and access employed by the MedUni Wien Biobank facility, and thus serve as a reference for research collaborators and investigators using this resource.

## Materials and Methods

### Biobank description

The MedUni Wien Biobank exists as a cross-institutional project carried out by the Department of Laboratory Medicine, the Department of Pathology, and the Institute of Neurology. The prospective collection of fluid samples for research purposes is mainly performed at the Department of Laboratory Medicine (KILM); hence the present results are restricted to processes at MedUni Wien Biobank (KILM).

The MedUni Wien Biobank (KILM) is organized as a section of the Department of Laboratory Medicine and as part of a quality management system maintained by its host department (certified according to ISO 9001:2015). It currently harbors ∼2 million aliquots derived from >100,000 submissions, each collected within specific biobank cohorts. At present, the Biobank implements technical specifications on pre-examination processes for sample handling, as recently published by the Technical Committee 140 “*in-vitro* diagnostics” of the Comité Européen de Normalisation (CEN).^5^ On a national level, the MedUni Wien Biobank contributes to the Austrian Biobank consortium BBMRI.at (www.bbmri.at, funded by the Austrian Federal Ministry of Education, Science and Research, Grant No. 10.470/0016-II/3/2013), which is the national hub of the European research infrastructure BBMRI-ERIC.^6^ Within this consortium, the MedUni Wien Biobank coordinates the Quality Management work package, the goal of which is to harmonize quality efforts within the Austrian biobanking landscape and to establish a culture of mutual quality audits between cooperating biobanks.

### Ethical aspects

All biobank collections require assessment by the Ethics Committee of the Medical University of Vienna before the first submission. Preparation of the study protocol and Ethics Committee approval are the duties of the clinical collaborator. However, MedUni Wien Biobank (KILM) will support its cooperation partners with formulation of resource-specific chapters. If not otherwise specified by the Ethics Committee, informed consent is collected and documented by the recruiting facility (in most cases: clinical collaborator). If not defined within the initial ethics vote, each subsequent project utilizing the banked material again requires ethical review.

### Process overview

**FIG. 1.**
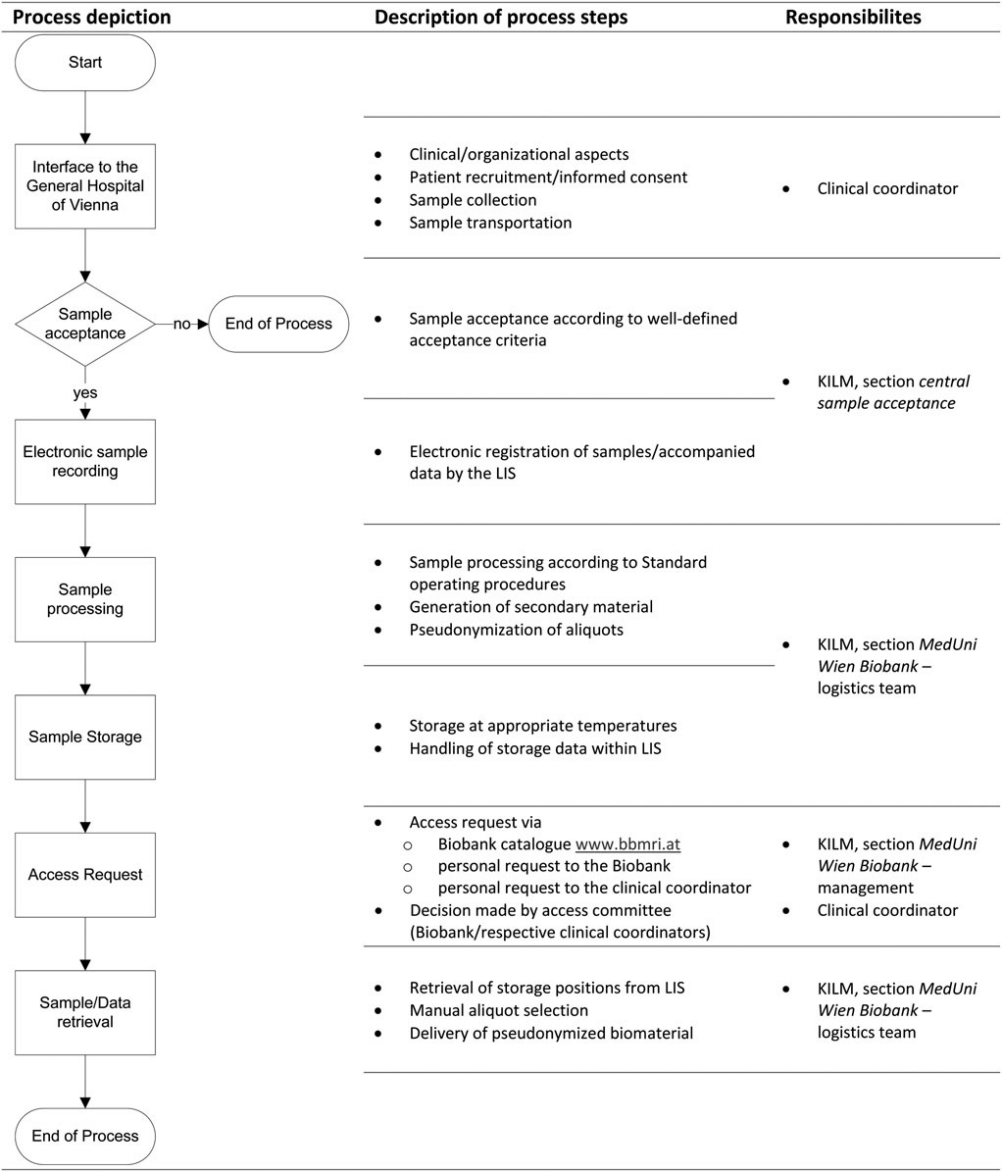
Process depiction for liquid sample banking at the MedUni Wien Biobank (KILM). KILM, Department for Laboratory Medicine; LIS, laboratory information system.

As required by the ISO 9001:2008 standard on quality management systems, the MedUni Wien Biobank has defined its core realization processes. A simplified visual description is shown in Figure 1. A detailed process description can be derived from the Supplementary Data (Supplementary Data are available online at www.liebertpub.com/bio).^5,7–10^

### Assessment of usage data

The annual numbers of stored samples and annual numbers of requested samples for research analyses were derived from the following databases: storage data were extracted from MOLIS (vision4health, Freienbach, Switzerland), which is the laboratory information and management system of the Department of Laboratory Medicine. Data on individual sample requests (requestor, number, type, purpose) are stored in a Microsoft Access-based database solution (Microsoft, Redmond, WA), which was created by the MedUni Wien Biobank (AccessStatistics v3) and is operated within a secure environment, and were exported to Microsoft Excel for further handling.

Publications arising from collaborating with the resource were assessed (1) by contacting the principal investigators that requested biobank material and, since only 9% of the collaboration partners responded, by (2) database research in PubMed on the name of the respective collaboration partner. Afterward, each article was examined and identified as having used the Biobank facility or not. Therefore, articles were screened for specific keywords identifying the collection (e.g., collection acronyms), or for the site of patient recruitment and sample withdrawal. Only original articles were taken into account; protocols or reviews were excluded. Impact factors of publications were derived from the Journal Citation Report^®^ (JCR^®^; Thomson Reuters, Toronto, Canada) valid on the day of article acceptance. It was assumed that the JCR^®^ of a specific year was published on July 1st of the following year, for example, JCR^®^ 2016 was used for publications accepted after July 1st, 2017.

Virtual costs per requested aliquots were calculated by dividing the number of requested aliquots by the sum of estimated biobanking costs for the same year. The latter comprised (1) the number of needed freezers +10% standby capacity, if each freezer provides space for 72,000 aliquots, (2) the approximate costs for two technical full-time equivalences according to the Austrian University Collective Labour Agreement level IIIa (∼€38,000 p.a.), and (3) costs for consumables and storage containers at €0.6 per stored aliquot. As preanalytical sample handling is widely integrated into routine workflows, no additional costs could be reasonably calculated to account for sample pipetting and centrifugation equipment. Energy costs were not taken into account.

### Statistical analysis

Full figures are given for stored/requested samples, resulting publications/impact factor points, and costs. Where appropriate, quotients are presented as percentages. Developments over time were calculated between categorically scaled time (years numbered from 1 to 8) and the metric variable of interest according to Spearman, and given as *ρ*. *p*-Values <0.05 were considered statistically significant. All calculations were performed in SPSS v24 (IBM, Armonk, NY). Graphical depictions were drawn in GraphPad Prism 6.07 (GraphPad, La Jolla, CA).

## Results

### Increasing access to fluid biomaterial

**FIG. 2.**
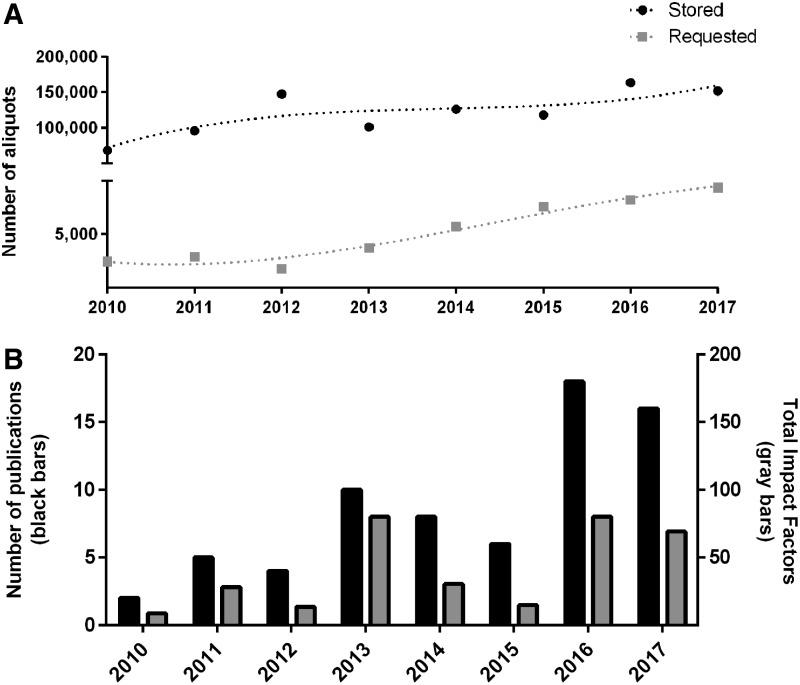
**(A)** Number of stored (*black dots*) and requested (*gray squares*) aliquots. **(B)** Number of original articles (*black bars*, *left y*-axis) and total annual impact factors (*gray bars*, *right y*-axis) that could be identified by database search, to which MedUni Wien Biobank (KILM) contributed.

As stated above, biobanking activities started in 2006, when the first prospective collections were established. Numbers of annually stored and accessed aliquots are given in Figure 2A. However, the first years until the end of the decade were regarded as a development phase and are hence excluded from this report. Changes over time were assessed by Pearson's correlation analyses.

**FIG. 3.**
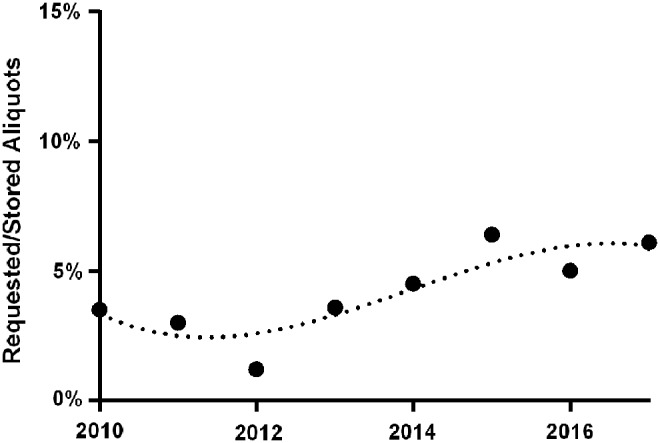
Quotient of requested to stored aliquots. The temporal course describes a sigmoid curve (*dotted curve*).

Numbers of processed and stored aliquots did significantly change over time (*ρ* = 0.810, *p* = 0.015). However, statistical significance is no longer present if only developments since 2012 are taken into account (*ρ* = 0.543, *p* = 0.266). In contrast, the number of stored aliquots requested for inclusion in research analyses presented with a steady increase over time (*ρ* = 0.929, *p* = 0.001). In 2010, access was requested for 2401 aliquots, and this amount constantly increased to 9342 aliquots in 2017. The quotient of requested to stored aliquots followed a sigmoid curve, with a minimum reached in 2012 (1.3% of the amount of stored samples were accessed, see Fig. 3). On average, ∼95% (e.g., 2016: 94.2% and 2017: 96.0%) of the requested aliquots could finally be delivered, while the remaining 5% were unavailable (e.g., no material left and inappropriate material left).

Hence, it appears that the usage of our biobank increased during the present decade and that the annual quotient between requested and stored samples settled at ∼6%. In a next step, we assessed how this was reflected by publication output.

### Publication output is not easy to assess, but rises with sample access rate

Publications arising from using the resource were intended to be registered via direct contact with biobank users. However, distribution of survey forms led to a poor questionnaire response rate. Hence, database searches based on the names of the principal investigators were performed as described in Materials and Methods section. Developments regarding the annual numbers of publications and total impact factors are given in Figure 2B.

The total number of biobank-associated publications was 69, resulting in a cumulative impact factor of 324.4. A total of 81% of the articles were published by the principal investigator collecting the samples, and a further 7% were published by other researchers of the Medical University of Vienna. About 10% of the articles emerged from a multicentric setting including the principal investigator, and only 1% to 2% of the articles were authored by external researchers. This distribution was similar to distribution observed among sample requestors. The annual number of publications increased between 2010 and 2017 (*ρ* = 0.857, *p* = 0.007) and the yearly amount of publications correlated with the quantity of requested samples (*ρ* = 0.857, *p* = 0.007).

Therefore it can be assumed that access to banked samples was converted to scientific output in the form of published articles. In a final step, incurred costs per requested sample were estimated.

### Virtual costs per accessed sample depend on access rate

As outlined above, academic, disease-oriented sample banking resulted in a considerable excess production of banked aliquots (e.g., in 2017, ∼16 times the amount of aliquots requested were stored). Therefore, we estimated virtual costs per requested sample as described in Materials and Methods section.

**FIG. 4.**
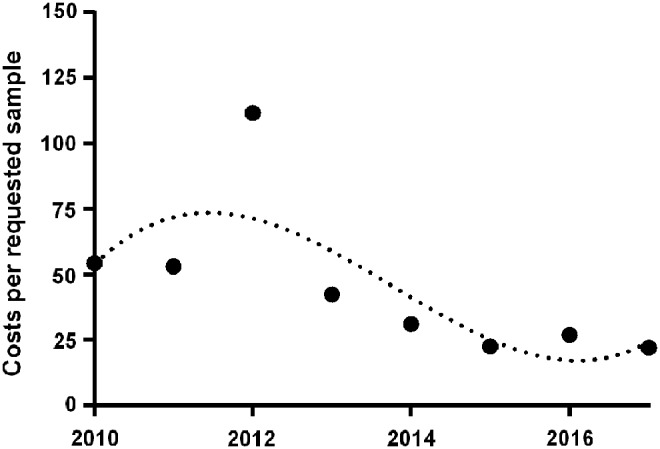
Virtual costs in € per accessed samples, consisting of expenses for personnel, storage devices, storage containers, and consumables for the number of stored aliquots, divided by the number of requested aliquots during the same year. The *dotted curve* shows the fitting curve for virtual cost development.

As the degree of overproduction decreased and the quotient of accessed aliquots to stored aliquots finally settled at 6% incurring costs per requested sample declined from >€50 to ∼€20 (Fig. 4).

## Discussion

The impact of standardized biobanks on biomedical research is considered to be high; however, actual data regarding the scientific output of academic biobanks are sparse. Hence we sought to report usage data of the academic, disease-oriented MedUni Wien Biobank regarding prospectively collected fluid samples to show what could be achieved in a large university hospital. Indeed, our data implied that usage of biomaterial as measured by absolute numbers of requested aliquots increases with the size of the resource. Of course, this went along with a considerable overproduction of banked samples. Since 2015, the degree of overproduction has leveled off at ∼15 to 20 times the amount of used aliquots. The virtual costs per requested aliquots declined as the usage rate increased and are currently a little over €20 per aliquot.

Reliable data on biobank usage are rare. This could be due to the fact that a high proportion of biobanks were established after the turn of the millennium^2^ and might thus still be under development. The MedUni Wien Biobank (KILM) was established in 2006, and data were reported from 2010 on. As our data show, numbers of annually stored aliquots continued to rise until 2012, which was 6 years after foundation, when a plateau was reached at ∼150,000 stored aliquots per year. The amount of requested samples is still on the rise, indicating that the usage of the resource might still have further potential. For other European biobanks, there is only one publication available regarding the usage of a blood donor biobank at a French blood transfusion center, reporting as little as stored 0.025% of samples requested. However, this biobank did obviously archive biomaterial for diagnostic or hemovigilance purposes and was not accessed for research requests.^11^ Hence, the present data could be highly valuable for academic institutions planning to develop a disease-oriented biobank, or to enable established biobanks to compare their performance.

Assessment of publication output was difficult, since cooperation partners rarely responded to the questionnaire provided. Hence, the overview of publications using the resource might not be complete. This is indeed an issue, as increasing availability of professional sample processing and storage facilities aroused publishers' and researchers' interest in the control of preanalytical conditions in the framework of biomedical investigations.^12–20^ At the same time, biobanks are required to track studies that have used their resources to assess their scientific or quality purposes.^3,21^ In recent years, transnational projects, for example, the Bioresource Research Impact Factor initiative funded by the European Commission, have addressed this issue and suggested referencing to each bioresource used within the Materials and Methods section of a research article.^22^ We hence decided to add detailed data on our biobank's sample processing workflows as Supplementary Data to the present article to generate a citable reference for biobank users that can be further traced within publication databases. Although the number of publications is generally on the rise, the curve follows a somehow undulating course. A cyclical reason cannot be ruled out, since low points of the curve are preceded by years of lower economic growth.^23^

Since overproduction declines with increasing access, virtual costs per requested aliquots dropped to slightly above €20 when personnel, storage devices, consumables, and storage containers were taken into account. These estimated costs are very moderate when compared to data provided by Clément et al.^24^ However, the figures might not be directly comparable, since Clément et al.^24^ included in their calculations a greater number of factors that were mainly administrative and supportive. Nevertheless, it can be assumed that MedUni Wien Biobank managed to decrease the virtual costs per requested aliquot as a result of the increasing access rates. Approximately 90% of all identified articles arose from the group that also collected the respective biomaterial. In contrast, external access was considerably low. Hence, minimization of overproduction might require the reinforcement of external collaborations.

It can be seen as a limitation that most publications using samples of the MedUni Wien Biobank were not reported by their principal investigators, but identified in the framework of literature research. Therefore, formal proof that the materials used did indeed originate from our biobank was not provided. Nevertheless, great care was taken when publications were screened for collection-specific identifiers or recruitment sites to avoid incorrect assignments.

In conclusion, the MedUni Wien Biobank (KILM) represents a growing and more intensively used resource mainly consisting of disease-oriented collections of fluid biomaterials. This bioresource has already contributed to considerable scientific output at a cost-effective level. However, especially the latter is not easy to assess in the absence of citable references for the biobank resources used. In conclusion, the reported figures might generate a basis for comparison for other academic, hospital-based biobanks.

## Supplementary Material

Supplemental data
