# Supplementary material for: Usage Data and Scientific Impact of the Prospectively Established Fluid Bioresources at the Hospital-Based MedUni Wien Biobank
Source: Biopreserv Biobank. 2018 Dec 17;16(6):477–82. doi: 10.1089/bio.2018.0032 (PMC6308288; doi:10.1089/bio.2018.0032)
Supplement: Supplemental data [file Supp_Data.pdf]

# Supplementary Data

## Detailed Process Description of MedUni Wien Biobank (KILM)

### *Interface to the General Hospital/Responsibilities of the clinical coordinator*

Each patient sample is collected within the framework of a particular biobank collection/study (rheumatology sample collection, kidney transplantation cohort, etc.). These collections/studies are designed and founded by principal investigators, who act as primary contractual partners of the biobank within the respective studies and are further referred to as clinical coordinators. Clinical coordinators bear responsibility for organizational, financial, ethical, and legal aspects of a study and act further—with regard to quality management—as the primary interface to the Quality Management System of the General Hospital of Vienna, in which patient contact, withdrawal of body fluids, and transportation of primary samples take place. Patient contact and sample withdrawal is performed by qualified medical personnel. Material dedicated for biobank storage is usually taken in the context of routine blood draws, whereby distinct sample tubes and submission forms are used. To ensure the quality of samples the MedUni Wien Biobank does not process and store left-over material from the diagnostic process. Each set of samples belonging to the same blood draw of a single individual is linked to a biobank-specific submission form. On the submission form, (material-specific) sample labels containing unique, barcoded job numbers are provided, enabling linkage between a single submission and all related samples. Moreover, the form should contain additional information about sample identification, date, and time of sample withdrawal and a collection-specific identifier (''project number'') (Supplementary Fig. S1).

Samples are subsequently transmitted to the Department of Laboratory Medicine by the sample transportation service (manual transport, automated transportation tracks, or pipe mail) of the General Hospital of Vienna.

### *Sample acceptance/registration*

Upon their arrival at the Department of Laboratory Medicine, the central sample receiving section evaluates the samples/data sheets according to the following rejection criteria: (1) the submission form must be complete and at least contain the patient's ID and the project number; (2) labels on specimens and submission forms must have corresponding job numbers to ensure correct linkage of samples and submission; (3) all sample containers must be correctly labeled with labels provided with the submission forms; and (4) macroscopic evidence of poor sample quality (incorrect container, insufficient sample quantity, obvious contamination of container). For samples meeting any of the rejection criteria the ordering physician will be contacted to clarify or to correct preanalytical mistakes. If correction is not possible the samples will be discarded or sent back to study coordinators.

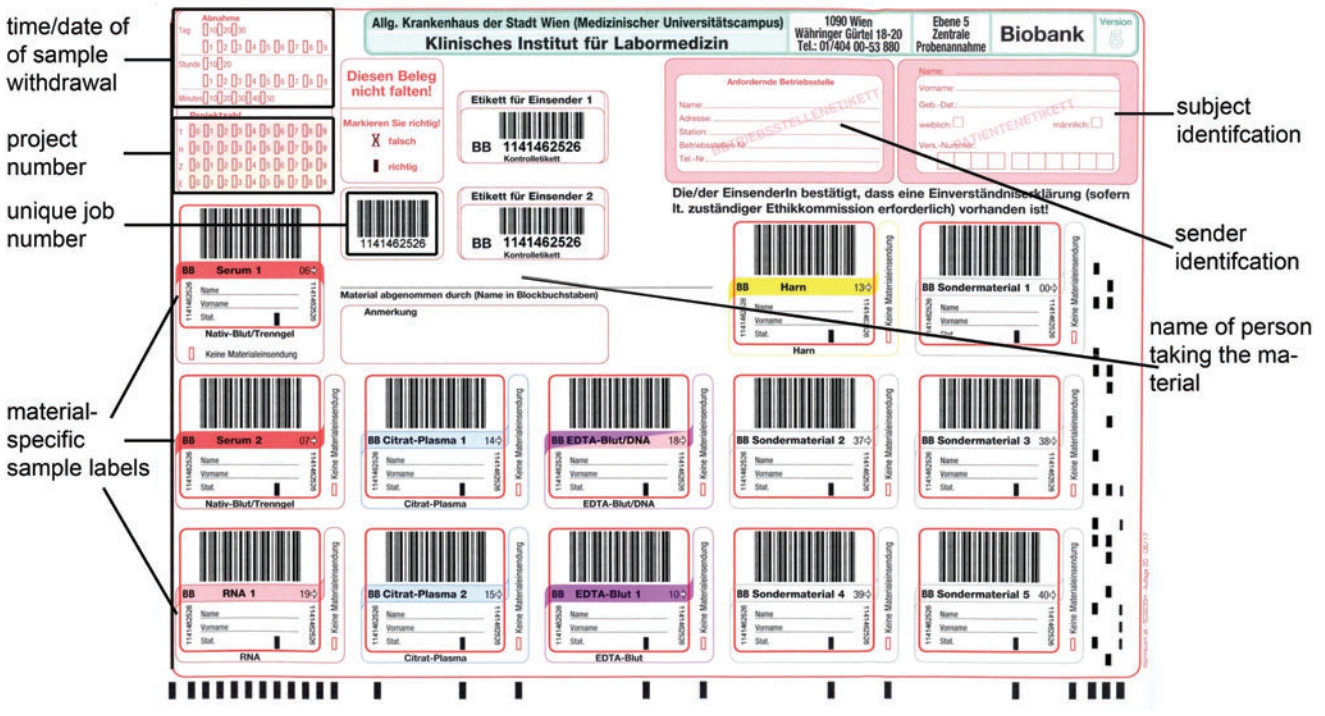


**SUPPLEMENTARY FIG. S1.** Biobank-specific submission form with unique job number and related sample labels, in compliance with the requirements of the technical specifications published by the CEN/TC 140.[S1]

Samples passing the receiving inspections are electronically registered. To this end, submission forms and barcode-labeled sample tubes are batch scanned into the laboratory information and management system (LIMS) MOLIS (vision4health, Bochum, Germany), creating a new dataset identified by the unique job number derived from the submission form/sample labels. Within this dataset, the project number (entered on the submission form by the sender) defines the required sample processing and storage conditions. Biospecimens are then transferred to the cobas p471/p512 pre-analytical system (Roche Diagnostics, Rotkreuz, Switzerland).

### Sample processing

The fully automated cobas p471/p512 pre-analytical system (Roche Diagnostics) allocates each biospecimen according to its downstream procedures; the platform communicates with the LIMS and requests sample-specific specifications. Samples that will undergo standard centrifugation conditions are thereby separated from samples that require different treatment (collection-specific, e.g., urine acidification). Standard samples are subsequently automatically centrifuged and aliquoted into 2D-barcoded polypropylene tubes on cobas p671/p612 pre-analytical devices (Roche Diagnostics), and special samples are manually processed by trained biomedical personnel (MedUni Wien Biobank logistics team). Standard handling conditions are given in Supplementary Table S1 in terms of SPREC (standard preanalytical codes) v2.0, as suggested by the International Society for Biological and Environmental Repositories.[S2]

### Isolation of secondary materials

Currently, the MedUni Wien Biobank offers prestorage isolation of genomic DNA and preparation of peripheral blood mononuclear cell (PBMC) lysates for later RNA isolation, and plasma storage for later extraction of circulating cell-free DNA.

PBMC lysates are produced from up to 25 mL $K_{2,3}$EDTA-anticoagulated whole blood (1:1 v/v diluted with phosphate-buffered saline) by means of Ficoll-based density-gradient centrifugation with porous separation barriers using commercially available cell separation tubes (Leucosep™; Greiner Bio-One, Kremsmuenster, Austria)[S3] according to the manufacturer's instructions. As a final step, the washed PBMCs are lyzed and stored in a guanidine isothiocyanate-containing buffer (Buffer RLT; Qiagen, Hilden, Germany) at ≤70°C until RNA isolation.

For later isolation of RNA and circulating cell-free DNA, commercially available collection tubes are used according to the manufacturers' instructions. If DNA extraction is required before storage, this is accomplished with up to 10 mL EDTA-anticoagulated whole blood using commercially available, precipitation-based kits according to the manufacturer's instructions. DNA concentration is assessed according to Warburg and Christian[S4] and is electronically documented. DNA purity is estimated by A260/280, whereby an A260/280 ratio of $1.8 \pm 0.2$ is accepted as sufficiently pure DNA.

### Storage

Serum, plasma, urine, cerebrospinal fluid, and secondary materials (genomic DNA, PBMC lysates) are transferred to

SUPPLEMENTARY TABLE S1. STANDARD PROCESSING CONDITIONS FOR LIQUID BIOSPECIMENS

| *Material* | *Serum* | *$K_2$, $K_3$ EDTA-plasma* | *Li heparin-plasma* | *$Na_3$ citrate-plasma* | *$K_2$, $K_3$ EDTA-whole blood* | *Urine* | *Cerebrospinal fluid* | *Stool* |
|---|---|---|---|---|---|---|---|---|
| SPREC | | | | | | | | |
| Material code | SER | PL1 | PL1 | PL1 | BLD | URN | CSF | STL |
| Type of primary container | SST (± separator gel) | EDG (± separator gel) | HEP | SCI (3.8% or 3.2% v/v) | PED | ZZZ (PET tube sterile) | PPS (PP tube sterile) | PPS (PP tube sterile) |
| Precentrifugation delay | A (<2 hours) | A (<2 hours) | A (<2 hours) | A (<2 hours) | — | A (<2 hours) | A (<2 hours) | — |
| Centrifugation | B 1884 × g, 10 minutes, RT | B 1884 × g, 10 minutes, RT | B 1884 × g, 10 minutes, RT | B 2499 × g, 15 minutes, 15°C | — | B 1884 × g, 10 minutes, RT | B 400 × g, 10 minutes, RT | — |
| Postcentrifugation delay | D (<2 hours RT) | D (<2 hours RT) | D (<2 hours RT) | D (<2 hours RT) | F (<8 hours RT) | D (<2 hours RT) | B (<1 hours RT) | D (<2 hours RT) |
| Long-term storage | S (2D barcoded PP tube, ≤70°C) | S (2D barcoded PP tube, ≤70°C) | S (2D barcoded PP tube, ≤70°C) | S (2D barcoded PP tube, ≤70°C) | Y (primary PET tube, ≤20°C) | S (2D barcoded PP tube, ≤70°C) | S (2D barcoded PP tube, ≤70°C) | Y, S [coded] PP tube, ≤20°C or ≤70°C) |

±, with or without; PP, polypropylene; RT, room temperature (18–25°C); SPREC, standard preanalytical codes.

2D-barcoded polypropylene tubes in aliquots up to 600 μL. The tubes are arranged in 96-well racks that allow for individual removal of aliquots. Storage occurs at a mean temperature of ≤70°C in standalone ultra-low freezing units. $K_{2,3}$-EDTA-anticoagulated whole blood for poststorage isolation of DNA is stored within the primary polyethylene terephthalate container at ≤20°C. Depending on the respective biobank collection, stool samples are either stored in the primary container (polypropylene) at ≤70°C or transferred to 2D-barcoded polypropylene tubes (filling volume up to 2 mL) in a 4×6 format. Of the latter, aliquots are stored at ≤70°C (and in some collections additionally at ≤20°C).

The influence of repeated storage temperature changes, inherent to single-freezer-based biobanks with repeated opening and closing of freezer doors, on analyte stability has been evaluated by the MedUni Wien Biobank and considered as insignificant for a broad range of markers.[S5]

2D-barcodes and storage positions are managed within the laboratory information system and can be easily requested upon sample access either for individual submissions (providing job number or sample identification) or in bulk via a database query (performed by the local IT service unit).

### *Access*

Access can be requested either through direct contact with the MedUni Wien Biobank management/the clinical coordinator of the respective collective, or via the national Biobank catalog available at www.bbmri.at. In general, access can only be granted in the context of collaborative scientific projects that conform to current ethical guidelines (e.g., Good Scientific Practice Guidelines of the Medical University of Vienna, Declaration of Helsinki) and have been approved by an Institutional Review Board/Ethics Committee. Thus, applicants have to provide information about their planned investigations (minimum requirements: study protocol, decision/approval of review board). Subsequently, an access committee created on an *ad hoc* basis, consisting of a Biobank representative and the respective clinical coordinator, consensually decides upon access. The Biobank reserves the right to demand appropriate financial compensation. The access procedures are further described on the Biobank's website (www.biobank.at).

## Supplementary References


S1. CEN. CEN/TC 140—In vitro diagnostic medical devices. 2017. Available at: https://standards.cen.eu/dyn/www/f?*p* = 204:7:0::::FSP_ORG_ID:6122&cs = 1E6FE477DD1CD53BDDE5FF10BAA8ACFB3. Accessed November 22, 2017.

S2. Lehmann S, Guadagni F, Moore H, et al. Standard preanalytical coding for biospecimens: Review and implementation of the Sample PREanalytical Code (SPREC). Biopreserv Biobank 2012;10:366–374.

S3. Hamot G, Ammerlaan W, Mathay C, et al. Method validation for automated isolation of viable peripheral blood mononuclear cells. Biopreserv Biobank 2015;13:152–163.

S4. Warburg O, Christian W. Isolation and crystallization of the fermentation enzyme enolase [German: Isolierung und kristallisation des gärungsferments enolase]. Naturwissenschaften 1941;29:589–590.

S5. Haslacher H, Szekeres T, Gerner M, et al. The effect of storage temperature fluctuations on the stability of biochemical analytes in blood serum. Clin Chem Lab Med 2017;55:974–983.
